# Psychometric properties of the Spanish version of the mindful attention awareness scale (MAAS) in patients with fibromyalgia

**DOI:** 10.1186/1477-7525-11-6

**Published:** 2013-01-14

**Authors:** Ausias Cebolla, Juan V Luciano, Marcelo Piva DeMarzo, Mayte Navarro-Gil, Javier Garcia Campayo

**Affiliations:** 1 Departamento de personalidad, evaluación y tratamientos psicológicos, Universitat Jaume I, CIBEROBN Ciber Fisiopatologia de la obesidad y la nutrición, Castellón, Spain; 2 Parc Sanitari Sant Joan de Déu and Fundació Sant Joan de Déu, Sant Boi de Llobregat, Barcelona, Spain; 3 Department of Family Medicine, University of Sao Paulo, Sao Paulo, Brazil; 4 Bitbrain Technologies, Zaragoza, Spain; 5 Department of Psychiatry, Miguel Servet Hospital & University of Zaragoza, Instituto Aragonés de Ciencias de la Salud, Red de Actividades Preventivas y de Promoción de la Salud (REDIAPP), Zaragoza, Spain

**Keywords:** Mindfulness, MAAS, Reliability, Validity, Fibromyalgia

## Abstract

**Background:**

Mindful-based interventions improve functioning and quality of life in fibromyalgia (FM) patients. The aim of the study is to perform a psychometric analysis of the Spanish version of the Mindful Attention Awareness Scale (MAAS) in a sample of patients diagnosed with FM.

**Methods:**

The following measures were administered to 251 Spanish patients with FM: the Spanish version of MAAS, the Chronic Pain Acceptance Questionnaire, the Pain Catastrophising Scale, the Injustice Experience Questionnaire, the Psychological Inflexibility in Pain Scale, the Fibromyalgia Impact Questionnaire and the Euroqol. Factorial structure was analysed using Confirmatory Factor Analyses (CFA). Cronbach's α coefficient was calculated to examine internal consistency, and the intraclass correlation coefficient (ICC) was calculated to assess the test-retest reliability of the measures. Pearson’s correlation tests were run to evaluate univariate relationships between scores on the MAAS and criterion variables.

**Results:**

The MAAS scores in our sample were low (M = 56.7; SD = 17.5). CFA confirmed a two-factor structure, with the following fit indices [sbX2 = 172.34 (p < 0.001), CFI = 0.95, GFI = 0.90, SRMR = 0.05, RMSEA = 0.06. MAAS was found to have high internal consistency (Cronbach’s α = 0.90) and adequate test-retest reliability at a 1–2 week interval (ICC = 0.90). It showed significant and expected correlations with the criterion measures with the exception of the Euroqol (Pearson = 0.15).

**Conclusion:**

Psychometric properties of the Spanish version of the MAAS in patients with FM are adequate. The dimensionality of the MAAS found in this sample and directions for future research are discussed.

## Background

In the last 20 years, an increasing number of studies have been dedicated to research on mindfulness and the use of mindfulness training as a clinical intervention for diverse physical and mental disorders. Mindfulness refers to an awareness that emerges by paying attention to purpose and to the present moment and non-judgmentally focusing on the unfolding of one’s immediate experience [1,2]. Mindfulness is a skill that can be taught using several uniquely designed techniques [3].

Mindfulness-based therapies have been demonstrated to be effective for the treatment of many disorders, including chronic pain conditions [4-6]. The mechanisms underlying the effects that mindfulness training has on health are diverse and include increased attention control, increased awareness of inner experiences, increased emotional regulation, and changes in the concept of self or in body awareness [7].

Mindfulness training in the treatment of fibromyalgia (FM) has been shown to decrease pain symptoms and to improve overall quality of life; as such, mindfulness training is considered a promising supplement to current interventions [4,8-10]. Despite these findings, there is still a lack of understanding of the mechanisms that underlie the mitigating effects of mindfulness on pain symptoms. Research studies on such mitigating effects suggest that mindfulness alters the contextual evaluation of pain [5], reduces pain catastrophising and pain sensitivity [6], reduces psychopathological symptoms [11,12], and alters pain-related anxiety [13]. These results have not been contradicted in the three years since their discovery [9].

Recent findings suggest that pain acceptance, which is promoted by mindfulness interventions, improves functioning and life quality. However, there is still a lack of reliable and valid instruments to assess relevant processes in such interventions [14]. It is assumed that if mindfulness is a learned skill, then a measure of mindfulness should demonstrate both incremental validity [15] and sensitivity to change. Furthermore, the expected changes (for example, improvement in quality of life or decrease in symptoms) should be directly related to changes in mindfulness.

There are several questionnaires that measure mindfulness, with the two the most commonly used being the Five-Facets Mindfulness Questionnaire (FFMQ) [16] and the Mindful Attention Awareness Scale (MAAS) [17]. The FFMQ is considered one of the most complete questionnaire because it measures five component skills of mindfulness: observing, describing, acting with awareness, nonjudging of inner experience and nonreactivity to inner experience. However, the MAAS is the most popular scale measuring mindfulness, with over 350 citations in the Web of Science [18]. The MAAS has shown theoretically consistent relationships to brain activity [19], treatment outcome in mindfulness-based interventions (MBIs) [20] and mediation of targeted MBI outcomes [21].

The authors that developed the MAAS define mindfulness as “the presence or absence of attention to, and awareness of, what is occurring in the present moment”. The MAAS is a 15-item scale developed to measure the frequency of mindful states in daily life. Translated variants of this scale have been validated in several languages, including Spanish [22], Chinese [23], Swedish [24], Turkish [25] and French [26].

The original Spanish scale was validated and developed in a sample of Spanish non-clinical participants. [22]. Within the clinical population, the MAAS has been validated only in a sample of cancer patients [27]. The validation of scales in specific clinical samples is important for research on mindfulness due to the recognised need for using valid measures in the assessment of interventions. In a recent study, the Five Facets of Mindfulness Inventory was validated in a sample of patients diagnosed with fibromyalgia. The results from this study showed that the data taken from the patient sample had a similar factorial structure to data taken from a healthy sample [28]. The purpose of the present study is to examine the psychometric properties of the Spanish version of the MAAS in a sample of patients diagnosed with fibromyalgia.

## Method

### Sample and procedure

Sample size was calculated according to the recommended 10:1 ratio of the number of subjects to the number of test items [29]. Participants were recruited from the Pain Clinic (Santander, Spain) and the Fibromyalgia Unit of the Miguel Servet Hospital, Zaragoza (Spain). Recruitment took place during the year 2010. To be included in the study, patients had to be diagnosed with fibromyalgia by a rheumatologist according to American College of Rheumatology (ACR) criteria [30]. Patients were excluded if they had a medical or psychiatric disorder that impeded their ability to correctly answer the questionnaire. The sample consisted of 251 participants (10 men and 241 women), with a mean age of 52.4 years (SD = 8.4; Range = 31–70). One patient was excluded as a result of being diagnosed with schizophrenia, which, in the clinician’s point of view, limited the reliability of the questionnaire. On average, participants had suffered from FM for 7.9 years (SD = 2.3; range = 1–20), and 122 participants (48.8%) had been granted an invalidity pension. The majority of patients (N = 231; 92.4%) were taking one or more prescription drug. More than half of the patients (N = 131; 52.4%) suffered from some form of psychiatric morbidity, as assessed by the MINI Psychiatric Interview [31] (mainly depression and anxiety). A group of 21 patients (8.4%) were also diagnosed with Posttraumatic Stress Disorder. The study questionnaires and protocol were approved by the Ethical Committee of the regional health authority, and patients signed a consent form attesting to their willingness to participate in the study.

### Instruments

#### Mindful attention awareness scale (MAAS)

The MAAS is a 15-item instrument measuring the general tendency to be attentive to and aware of one’s experiences in daily life [17]. Using a 6-point Likert-type scale (ranging from almost always to almost never), respondents rated how often they experienced acting as if they were on automatic pilot, being preoccupied, and not paying attention to the present moment (e.g.: “I could be experiencing some emotion and not be conscious of it until some time later”).The scale showed an internal consistency of 0.82 and exhibited significant convergent and discriminant validity. Scores on the MAAS were significantly higher in mindfulness practitioners than in matched community controls. The Spanish version of the MAAS was used, and it has recently been shown to have good test-retest reliability and internal consistency in a sample of healthy Spanish subjects [22].

#### Chronic pain acceptance questionnaire (CPAQ)

The CPAQ is a 20-item questionnaire designed to measure pain acceptance (e.g.: “It’s OK to experience pain”) [32]. All items are rated on a Likert-type scale, ranging from 0 (never true) to 6 (always true). The Spanish version of the scale has been validated [33], showing sufficient test-retest reliability and internal consistency.

#### Pain catastrophizing scale (PCS)

The PCS is a 13-item scale designed to assess individuals’ catastrophising cognitions by asking them to reflect on thoughts or feelings associated with present painful experiences (e.g.: “When I’m in pain I feel I can’t go on”) [34]. Each item is scored on a Likert-type scale, which ranges from 0 (not at all) to 4 (always). The Spanish version of this scale has been validated, [35] showing sufficient test-retest reliability and internal consistency.

#### Injustice experience questionnaire (IEQ)

The IEQ is a 12-item questionnaire that measures the frequency with which patients have thoughts concerning the unfairness of their illness (e.g.: “My life will never be the same”) [36]. Each question is answered using a 5-point scale, which ranges from 0 (never) to 4 (all the time). The Spanish version of this scale has been validated, [37] showing sufficient test-retest reliability and internal consistency.

#### The psychological inflexibility in pain scale (PIPS)

The PIPS [14] is a 12-item scale developed to assess target variables in exposure and acceptance-oriented treatments of chronic pain (e.g.: “I postpone things because of my pain”). We used the total scores resulting from this instrument in the final analyses of this study. The Spanish version of PIPS has been validated by our group “(personal communication)”.

#### EuroQol (EQ-5D)

The EQ-5D is a questionnaire composed of 7 items developed to measure a unique health status score [38]. The EQ-5D covers 5 dimensions of health: mobility, self-care, usual activities, pain/discomfort and anxiety/depression. Each dimension is evaluated in 3 categories (no problem, moderate problems, or extreme problems). In the present study, we used a validated Spanish version of EQ-5D [39].

#### Fibromyalgia impact questionnaire (FIQ)

The Fibromyalgia Impact Questionnaire (FIQ) is a 10-item self-report questionnaire developed to measure the functional impairment of fibromyalgia patients [40]. The first item of the scale focuses on the patient's ability to carry out muscular activities. The next two items of the scale ask patients to indicate the number of days in the past week that they felt good and the number of instances that they missed work. Finally, the last seven items (i.e., ability to work, pain, fatigue, morning tiredness, stiffness, anxiety and depression) are measured with visual analogue scales. The Spanish version of this scale has been validated [41].

### Statistical analyses

Demographic data were analysed using the descriptive statistics of mean, standard deviation (SD) and range. Prior to conducting the statistical analyses, we examined data for univariate and multivariate outliers. In order to detect the presence of univariate outliers, the frequency distributions of each item was examined (values ≥ 3 standard deviations from the mean indicate univariate outliers). Screening for multivariate outliers was by carried out by means of the Mahalanobis distance scores for all cases (D2); A D2 probability ≤ 0.01 indicates the existence of multivariate outliers [42]. We did not detect any outliers, therefore all cases were retained for the statistical analyses.

We used Confirmatory Factor Analysis (CFA) to analyse the dimensionality of the MAAS. We propose a one-factor model (with all items loading on one latent factor) and a two-factor model (Factor 1: items 1, 2, 4, 5, 7, 8, 9, 10, 11, 14, and 15; Factor 2: items 3, 6, 12, and 13) previously found with a principal component analysis. EQS software for Windows version 6.1 [43] was used to conduct the CFA. The maximum likelihood with robust correction method was used to adjust for distributional problems in the data set. Although a model with a non-significant chi-square estimate is generally considered a model with good fit, Hu and Bentler [44] recommended combinational rules to evaluate model fit. Therefore, we analysed the following indices (values in parentheses denote goodness of fit standards): Comparative Fit Index and Goodness of Fit Index (CFI and GFI > 0.90) and Root Mean Square Error of Approximation and Standardized Root Mean-Square Residual (RMSEA and SRMR < 0.08). The Satorra–Bentler chi-square is a chi square fit index that corrects the statistic under distributional violations. To reduce the sensitivity of chi-square to sample size, the index is divided by the degrees of freedom. Ratios of 3 or smaller are indicative of an acceptable fit of the model [45]. We selected these statistics to measure fit because previous research corroborated their performance and stability [46].

We examined the internal consistency, test-retest, and construct validity of the MAAS. Cronbach's α coefficient [47] was used to analyse the internal consistency of the scale. Corrected item-total correlations, in which an item is correlated with the total scale score excluding itself, were tested for each item. Consistency of the MAAS total score over time (test-retest reliability) was assessed using the Intraclass Correlation Coefficient (ICC). Construct validity was examined by correlating the MAAS with theoretically related and unrelated constructs. Pearson’s correlations were performed to evaluate univariate relationships between the MAAS and the following criterion variables: chronic pain acceptance, pain catastrophising, perceived injustice, pain inflexibility, global function and quality of life. We used effect size criteria outlined by Cohen [48] to evaluate the substantive significance of correlations (i.e., large correlations are those >0.50, medium correlations range from 0.30 to 0.49, and small correlations range from 0.10 to 0.29).

## Results

All items were examined in terms of mean, standard deviation, skewness and kurtosis. Univariate values approaching at least 2.0 for skewness and 7.0 for kurtosis indicate marked non-normality [42]. On the basis of the values displayed in Table 1, the data appear to show normality.

**Table 1:** Means (M), standard deviation (SD), 95% Confidence Intervals (95% CIs), standardised factor loadings (λ one-factor solution), corrected item-total correlations (rtot), skewness and kurtosis for all MAAS items

**MAAS items (Spanish translation between parentheses)**	**M (SD)**	**95% CIs**	**λ**	**rtot**	**Skewness**	**Kurtosis**
1. I could be experiencing some emotion and not be conscious of it until some time later (Puedo estar experimentando alguna emoción y no ser consciente hasta algún tiempo después)	4.39 (1.6)	4.1-4.6	0.53	0.53	-.44	−1.22
2. I break or spill things because of carelessness, not paying attention, or thinking of something else (Rompo o derramo cosas por descuido, por no prestar atención o por pensar en otra cosa).	4.15 (1.7)	3.9-4.3	0.60	0.59	-.30	−1.38
3. I find it difficult to stay focused on what’s happening in the present (Encuentro difícil permanecer focalizado en lo que está ocurriendo en el presente).	3.34 (1.7)	3.1-3.5	0.60	0.59	.32	−1.13
4. I tend to walk quickly to get where I’m going without paying attention to what I experience along the way (Tiendo a andar rápidamente para llegar a donde quiero ir sin prestar atención a lo que experimento a lo largo del camino).	3.82 (1.8)	3.5-4	0.59	0.62	-.08	−1.50
5. I tend not to notice feelings of physical tension or discomfort until they really grab my attention (Tiendo a no notar la tensión física o el malestar hasta que realmente despierta mi atención).	4.61 (1.7)	4.4-4.8	0.44	0.42	-.83	-.82
6. I forget a person’s name almost as soon as I’ve been told it for the first time (Olvido el nombre de una persona casi tan pronto como me lo dicen por primera vez).	2.14 (1.6)	1.9-2.3	0.34	0.32	1.32	.37
7. It seems that I am “running on automatic pilot,” without much awareness of what I’m doing (Parece que lleve puesto el “piloto automático” sin ser consciente de lo que estoy haciendo).	3.31 (1.8)	3-3.5	0.81	0.76	.32	−1.29
8. I rush through activities without being really attentive to them. (Hago las actividades diarias corriendo sin estar realmente atento a ellas).	4.01 (1.7)	3.7-4.2	0.80	0.72	-.18	−1.46
9. I get so focused on the goal I want to achieve that I lose touch with what I’m doing right now to get there (Estoy tan centrado en la meta que quiero alcanzar que pierdo la noción de lo que estoy haciendo).	4.37 (1.8)	4.1-4.6	0.81	0.76	-.55	−1.28
10. I do jobs or tasks automatically, without being aware of what I'm doing (Hago tareas o trabajos automáticamente sin ser consciente de lo que estoy haciendo).	4.08 (1.8)	3.8-4.3	0.82	0.75	-.28	−1.47
11. I find myself listening to someone with one ear, doing something else at the same time. (Me encuentro a mí mismo escuchando a alguien mientras hago algo al mismo tiempo).	3.81 (1.7)	3.6-4	0.47	0.45	.03	−1.52
12. I drive places on “automatic pilot” and then wonder why I went there (Conduzco a sitios con el “piloto automático” y entonces me pregunto qué hago allí).	3.47 (1.9)	3.2-3.7	0.66	0.65	.14	−1.52
13. I find myself preoccupied with the future or the past (Me encuentro a mí mismo preocupado por el futuro o el pasado).	3.04 (1.8)	2.8-3.2	0.42	0.42	.50	−1.14
14. I find myself doing things without paying attention (Me encuentro a mí mismo haciendo cosas sin prestar atención).	3.68 (1.7)	3.4-3.9	0.80	0.76	.10	−1.39
15. I snack without being aware that I’m eating (Picoteo sin ser consciente de lo que estoy comiendo).	4.52 (1.9)	4.2-4.7	0.49	0.48	-.81	−1.01

As shown in Table 1, descriptive statistics were computed for all MAAS items. The mean total score on the MAAS was 56.7 (SD = 17.5; range 18–90). The highest score was obtained for item 5, which asks about the subject’s tendency not to notice feelings of physical tension and discomfort until these symptoms grab his or her attention. The lowest score was obtained for item 6, which asks about the tendency to forget the name of a person immediately.

### Confirmatory factor analysis (CFA)

The original one-factor model [16] showed good fit indices _sb_X^2^ = 185.43 (*p* < 0.001); CFI = 0.94; GFI = 0.89; SRMR = 0.05; RMSA = 0.07 (0.05-0.08)]. The two-factor model, based on a previous exploratory factor analysis, obtained slightly better fit indices _sb_X^2^ = 172.34 (*p* < 0.001), CFI = 0.95, GFI = 0.90, SRMR = 0.05, RMSEA = 0.06 [0.05-0.08]]. The factor loadings of all MAAS items are shown in Table 1.

### Reliability

Cronbach’s α for the MAAS was 0.90, indicating a high degree of internal consistency. Corrected item-total *r* correlation coefficients ranged between 0.32 and 0.76. With regard to temporal stability, a subsample of 162 patients from the original sample was randomly selected and contacted by phone in order to arrange a new interview to complete the instruments again 1–2 weeks later. This subsample included 5 men and 156 women, with a mean age of 50.8 years (SD = 7.9; Range = 33–68). Data from this subsample showed a test-retest coefficient of 0.90 (CI = 0.89–0.92).

### Construct validity

The convergent and divergent validity of the MAAS was calculated using Pearson’s product–moment correlations with other relevant measures of psychopathology and measures of level of acceptance related to pain (see Table 2). Overall, with the exception of the EQ-5D, the measures correlated moderately and significantly with total scores on the MAAS.

**Table 2:** Means (M) and standard deviations (SD) of study measures and association with MAAS total score in fibromyalgia patients

	**M (SD)**	**MAAS**
CPAQ	47.6 (23.3)	0.37^**^
PCS	24.3 (13.6)	−0.47^**^
FIQ	58.0 (15.0)	−0.46^**^
EIQ	30.1 (12.1)	−0.45^**^
PIPS	57.1 (18.2)	−0.47**
EQ-5D	47.1 (19.8)	0.15^*^

*p < 0.05; ** p < 0.001.

MAAS = Mindful Attention Awareness Scale; CPAQ = Chronic Pain Acceptance Questionnaire; PCS = Pain Catastrophizing Scale; FIQ = Fibromyalgia Impact Questionnaire; IEQ = Injustice Experience Questionnaire; PIPS = The Psychological Inflexibility in Pain Scale; EQ-5D = Health-related quality of life.

## Discussion

The main objective of this study was to examine the psychometric properties of the Spanish version of the MAAS in a sample of patients with fibromyalgia. The MAAS scoring in our sample of patient s with FM (N = 251; M = 3.78; SD = 1.68) compared with the community adults sample studied in the original validation study (17) (N = 436; M = 4.20, SD = .69) is significantly lower (*t* = −4.592; gl = 685; *p* <0.001). These data show a tendency of FM patients to be less aware of their experience in daily life, acting more on “autopilot” and paying less attention to the present moment than healthy population does. A descriptive analysis of the items and the total score showed a tendency of FM patients to be less aware of their experience in daily life, acting more on “autopilot” and paying less attention to the present moment than healthy population does.

The results found using CFA are largely consistent with those reported in previous studies [17,22-27,49]. In the current sample, the one- and two-factor models both show adequate fit; however, we decided to retain the one-factor model for the set of reasons outlined below. First, the one-factor model met all the pre-established fit criteria, except for the chi-squared goodness-of-fit statistic, which was statistically significant (an unsurprising result, given that this statistic is highly sensitive and even small differences in model fit are statistically significant). Second, with the exception of item 6, all items loaded strongly on the latent factor (all factor loadings exceeded 0.40). Third, the underlying construct of the second latent factor in the two-factor model is difficult to interpret, other than on the basis of the item difficulty of the 4 loaded items. For instance, forgetting another person’s name almost as soon as one has been introduced for the first time is quite common, even amongst healthy individuals; this item had the lowest mean score. The two-factor model was proposed on the basis of a previous exploratory factor analysis, and it is well known that “artifactual difficulty” factors may be generated in unidimensional instruments when using exploratory techniques [50]. Fourth, the one-factor structure of the MAAS gained further support from the internal consistency analysis, which yielded an excellent Cronbach's α. Fifth, all items showed a corrected item-total correlation that was higher than conventional minimum value of 0.20.

The test-retest reliability analysis yielded good temporal stability in a 1–2 week period. Regarding the correlation analyses, almost all of the measures included in the study correlated in the expected way with the MAAS total scores. These results are consistent with those found in other studies that demonstrate the importance of acceptance capacity in the experience of pain [5,6]. The only exception to this pattern of findings was the correlations between the MAAS and EQ-5D. However, these data are not surprising, given the results found by Boomershine [51], who performed a comprehensive evaluation of standardised assessment tools in the diagnosis of the fibromyalgia syndrome and in the assessment of fibromyalgia severity. In this evaluation, the EQ-5D was not among the recommended instruments for assessing HRQL or global improvement in these patients with FM.

The two-factor structure was best supported by the data found in this research study, but results are not strong enough to conclude that this factorial model is best for the reasons already described. In both cases (uni and bifactorial models), the factor structure exhibited an acceptable fits, although more research is needed to explore the stability of the factor structure in FM and other chronic pain patients.

This study has several limitations. First, as in any study using self-report measures, the results may have been influenced by participants’ acquiescence and need for social desirability. Furthermore, the validity of self-report measures of mindfulness, and the MAAS in particular, have been criticised previously [18]. One such criticism is that respondents might not be fully aware of their ability to experience the present moment. Second, we did not assess the instrument in populations of patients with other types of chronic pain, thus we did not confirm whether the factor structure is or is not specific to fibromyalgia. Third, the overwhelming proportion of women limits the generalizability of the findings to men. And finally, the difficulty in interpreting the confirmatory factor analyses warrants more research studies.

## Conclusion

In conclusion, the MAAS has been shown to be a reliable instrument for measuring mindfulness in fibromyalgia patients. The results found through the factor structure analyses in this study should be examined in future studies. Such studies may compare the current results with those taken from clinical samples suffering from other types of chronic pain.

## Abbreviations

FM: Fibromyalgia; MAAS: Mindful attention awareness scale; CFA: Confirmatory factor analysis; ICC: Intraclass correlation coefficient; SD: Standard deviation; ACR: American college of rheumatology; CPAQ: Chronic pain acceptance questionnaire; PCS: Pain catastrophizing scale; IEQ: Injustice experience questionnaire; PIPS: The psychological inflexibility in pain scale; EQ5D: EuroQol; FIQ: Fibromyalgia impact questionnaire; CFI: Comparative fit index; GFI: Goodness of fit index; RMSEA: Root mean square error of approximation; SRMR: Standardized root mean-square residual; CI: Confidence interval.

## Competing interest

The authors declare that they have no competing interests.

## Authors’ contributions

AC and JGC designed the project. MNG and MPDM collected the data. AC and JVL performed the statistical analysis, and all authors interpreted the results, drafted the manuscript and read and approved the final manuscript.
